# Electropolymerization of Donor–Acceptor Conjugated Polymer for Efficient Dual‐Ion Storage

**DOI:** 10.1002/advs.202310239

**Published:** 2024-04-06

**Authors:** Xianhe Chen, Weisheng Zhang, Chenxing Zhang, Yuxuan Guo, Ao Yu, Shilin Mei, Chang‐Jiang Yao

**Affiliations:** ^1^ State Key Laboratory of Explosion Science and Safety Protection School of Mechatronical Engineering Beijing Institute of Technology Beijing 100081 China

**Keywords:** bipolar cathode, high energy density, in situ polymerization, lithium‐ion battery, organic electrode

## Abstract

Rationally designed organic redox‐active materials have attracted numerous interests due to their excellent electrochemical performance and reasonable sustainability. However, they often suffer from poor cycling stability, intrinsic low operating potential, and poor rate performance. Herein, a novel Donor–Acceptor (D–A) bipolar polymer with *n*‐type pyrene‐4,5,9,10‐tetraone unit storing Li cations and *p*‐type carbazole unit which attracts anions and provides polymerization sites is employed as a cathode for lithium‐ion batteries through in situ electropolymerization. The multiple redox reactions and boosted kinetics by the D–A structure lead to excellent electrochemical performance of a high discharge capacity of 202 mA h g^−1^ at 200 mA g^−1^, impressive working potential (2.87 and 4.15 V), an outstanding rate capability of 119 mA h g^−1^ at 10 A g^−1^ and a noteworthy energy density up to 554 Wh kg^−1^. This strategy has significant implications for the molecule design of bipolar organic cathode for high cycling stability and high energy density.

## Graphical abstract

A Donor–Acceptor (D–A) bipolar polymer with *n*‐type pyrene‐4,5,9,10‐tetraone units and *p*‐type carbazole units for lithium‐ion batteries is achieved by in situ electropolymerization. The cathodes reach a high discharge capacity (202 mA h g^−1^), an outstanding rate capability (119 mA h g^−1^ at 10 A g^−1^), and a noteworthy energy density (554 Wh kg^−1^).

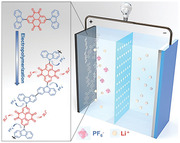

## 1 Introduction

With the rapid development of redox‐active organic electrode materials, the avenue toward green energy storage systems (EESs) has become broader than ever owing to their merits of elemental abundance, environmental benignity, structure/property diversity, and flexibility.^[^ ^1^ ^]^ Based on the redox reaction mechanism, organic electrode materials can be divided into three types: *n*‐type, *p*‐type, and bipolar‐type.^[^ ^2^ ^]^ The p‐type materials tend to lose electrons and accept anions, while *n*‐type materials operate by accepting electrons and combining with cations. Compared to *n*‐type materials, *p*‐type materials exhibit a higher operating voltage but lower specific capacity.^[^ ^3^ ^]^ Dual‐ion batteries (DIBs) have emerged as promising alternatives to conventional LIBs due to the merits of high working voltage, material availability, low cost, and excellent safety. Upon the dual‐ion storage mechanism, i.e., the simultaneous intercalation of anions into the cathode and cations into the negative electrode during the charging process, a variety of organic electrode materials including nitrogen‐ and sulfur‐containing redox active molecules, metal‐organic compounds, radicals, and polycyclic aromatic hydrocarbons have been explored for DIBs.^[^ ^4^ ^]^ However, the capacities of these *p*‐type materials tend to be relatively low, which constrains the energy density; whereas bipolar‐type materials are capable of utilizing both positively and negatively charged states, potentially combining the benefits (i.e., high capacity, high voltage, and fast kinetics) of both *n*‐type and *p*‐type parts to construct high‐performance DIBs.^[^ ^5^ ^]^ Nevertheless, previously reported bipolar‐type materials often suffer from low capacity, which can be attributed to the introduction of inactive units and relatively lower working potential when compared to *p*‐type materials. For example, Lu et al. prepared a novel bipolar polyimide COF (NT‐PICOF), which demonstrates the high capacity of 165 mA h g^−1^ at 30 mA g^−1^ and high capacity retention of 91% after 4000 cycles at 1 A g^−1^, but possess relatively low discharge voltage at ≈2.36, 3.54, and 3.93 V.^[^ ^5f^ ^]^

Pyrene‐4,5,9,10‐tetraone (PTO), with its four active carbonyl sites (*n*‐type), is a promising cathode material for lithium‐ion batteries due to its high theoretical capacity of 408 mA h g^−1^. Its intrinsic aromatic conjugated structure and fast kinetics make it a favorable component of organic cathode materials. Nonetheless, its dissolution in electrolytes and low conductivity can lead to capacity loss and poor performance. Among various approaches depressing the dissolution problem such as salification,^[^ ^6^ ^]^ modification with insoluble materials,^[^ ^7^ ^]^ polymerization,^[^ ^8^ ^]^ and regulating the electrolyte,^[^ ^9^ ^]^ in situ electropolymerization stands out in terms of high efficiency, low inactive segments and good conductivity of the generated electrodes.^[^ ^10^ ^]^ Therefore, it is expected to deliver high specific capacity, excellent cycling stability, and good rate performance. The carbazole (CZ) group stands out as a component of organic cathode materials, not only offering an active site (*p*‐type) for energy storage but also being capable of polymerization under electrochemical oxidation conditions, thereby making it an advantageous selection for enhancing cycling stability. It is worth noting that CZ, with its tertiary amine structure, possesses a smaller inactive mass compared to the same *p*‐type material such as triphenylamine. This reduced inactive mass of CZ is beneficial for enhancing the theoretical capacity of the polymer cathode. For instance, Xu's group reported the synthesis of a *p*‐type polymer 4,4′,4″‐Tris‐(carbazol‐9‐yl)‐triphenylamine (TCTA) via in situ electropolymerization.^[^ ^11^ ^]^ The TCTA cathodes showed a remarkable rate capability of 20 A g^−1^, and outstanding cycling stability (5000 cycles at 1A g^−1^), but a relatively low energy density of 365.2 Wh kg^−1^ due to the introduction of inactive moieties that sacrificed the specific capacity. Subsequently, another electropolymerized cathode amino‐phenyl carbazole naphthalene diimide (APCNDI) was developed with an improved energy density of 451.2 Wh kg^−1^.^[^ ^12^ ^]^ How to combine high working potential while maintaining high capacity, excellent rate capability and long cycling life is still challenging for organic cathodes.

Herein, we report a novel bipolar‐type cathode for rechargeable LIBs via in situ electropolymerization. The monomer 2,7‐di(9H‐carbazol‐9‐yl)pyrene‐4,5,9,10‐tetraone (PTO‐2CZ) consists of both *p*‐type carbazole (CZ) and *n*‐type pyrene‐4,5,9,10‐tetraone (PTO) that enable multiple redox reactions to combine high capacity with high working potential. The calculated HOMO and LUMO of PTO‐2CZ were located above the CZ and PTO unit, respectively, indicating that CZ and PTO act as the donor and acceptor, respectively. This D–A structure extends the π–π conjugation and promotes charge transfer inside the molecule, which endows the material with excellent rate performance.^[^ ^13^ ^]^ Significantly, the PTO‐2CZ monomer bears fewer inactive segments or moieties, which mitigates the sacrifice of the specific capacity. Furthermore, The electropolymerization behavior and reaction mechanisms of PTO‐2CZ were also investigated. The results reveal that the in situ electropolymerization not only can depress the severe dissolution but also lead to enhanced reaction kinetics that afford excellent electrochemical performance. As a result, the in situ electropolymerized PPTO‐2CZ cathode exhibits an exceptional discharge capacity (202 mA h g^−1^ at 200 mA g^−1^), a notable working potential (2.87 and 4.15 V), excellent rate performance (119 mA h g^−1^ at 10 A g^−1^) and remarkable energy density (554 Wh kg^−1^).

## 2 Results and Discussion

**Figure 1:**
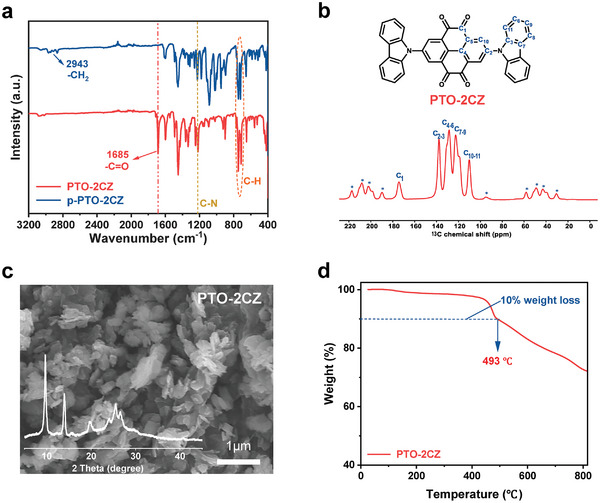
a) FTIR spectrum of p‐PTO‐2CZ and PTO‐2CZ. b) Molecule structure and Solid ^13^C NMR spectra of PTO‐2CZ. c) SEM image and XRD pattern of PTO‐2CZ, and (d) TGA spectra of PTO‐2CZ.

PTO‐2CZ was synthesized by Ullmann coupling with a yield of ≈50% (Schemes S1 and S2, Supporting Information). The obtained product was confirmed by Fourier transform infrared (FTIR) spectroscopy (**Figure** **1**a) and ^1^H and ^13^C nuclear magnetic resonance (NMR; Figure 1b; Figure S1, Supporting Information). Using a protection‐deprotection strategy, the tetrone‐protected precursor p‐PTO‐2CZ was first synthesized to avoid the irreversible consumption of active carbonyls in the product. Finally, the monomer PTO‐2CZ with recovered carbonyls was prepared by deprotection with trifluoroacetic acid (TFA) and water. In the FTIR spectrum (Figure 1a), p‐PTO‐2CZ displays apparent multiple peaks of the ─CH_2_ group at ≈2943 cm^−1^ and no typical carbonyl (C═O) peaks can be observed at ≈1680 cm^−1^, indicating the successful protection of carbonyl groups. As for PTO‐2CZ, the multiple peaks at ≈2940 cm^−1^ disappear and a strong peak at ≈1685 cm^−1^ appear because of the recovery of C═O functional groups after deprotection. Furthermore, the peak at ≈1225 cm^−1^ can be attributed to the C─N vibration of carbazole groups. The carbonyl groups of PTO‐2CZ and the C─N bonds of the carbazole groups are also represented by the ^13^C NMR spectrum's characteristic chemical shifts at 174 and 138 ppm, respectively (Figure 1b). Scanning electron microscopy (SEM) image and X‐ray diffraction (XRD) pattern (Figure 1c) suggest that the PTO‐2CZ monomer possesses a highly crystalline structure. Thermal gravimetric analysis (TGA) of PTO‐2CZ reveals that the decomposition temperature with 10% weight loss is 493 °C, suggesting strong thermal stability as active electrochemical materials in rechargeable batteries (Figure 1d).

**Figure 2:**
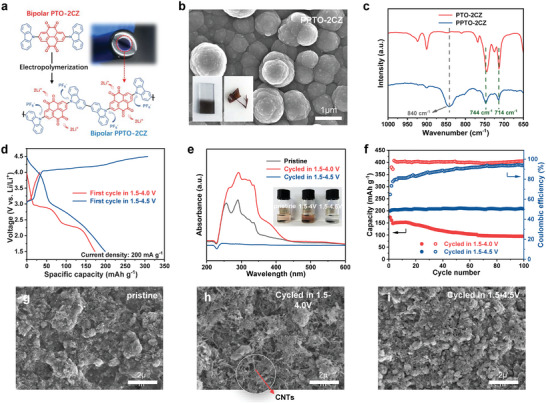
a) Schematic illustration of the electropolymerization of PTO‐2CZ and a digital image of electropolymerized PTO‐2CZ on a glass carbon electrode. b) SEM and digital images of PPTO‐2CZ. c) FTIR spectrum of PTO‐2CZ and PPTO‐2CZ. d) Galvanostatic charge/discharge profiles of PTO‐2CZ cathodes at different cut‐off voltages in the 1st cycle. (e) UV–vis spectra and digital images of pristine and cycled PTO‐2CZ cathodes in different voltage windows of 1.5–4.0 and 1.5–4.5 V. f) Cycling stability of PTO‐2CZ cathodes at different cut‐off voltages at a current density of 200 mA g^−1^. SEM images of g) pristine and cycled PTO‐2CZ cathodes in different voltage windows of h) 1.5–4.0 V and i) 1.5–4.5 V.

The electropolymerization of PTO‐2CZ was evaluated by the electrochemical test with a glass carbon electrode (GCE) as the working electrode in a solution of dry dichloromethane containing 3 mM PTO‐2CZ to verify the speculated in situ electropolymerization mechanism (**Figure** **2**a). CV test in the potential range of 0–1.6 V (vs Ag/Ag^+^) reveals typical polymerization characteristics of carbazole, and the shiny metallic surface of the working electrode becomes bluish violet, indicating the formation of electropolymerized product (Figure 2a; Figure S2, Supporting Information). When indium tin oxide (ITO)‐coated glass was used as the working electrode, the CV curve was similar to that of the glass carbon (Figure S3a, Supporting Information). Interestingly, a freestanding dark brown polymer film can automatically peel off the ITO substrate after a number of cycles (Figure 2b, inset). The obtained PPTO‐2CZ is composed of uniform spherical particles with an average diameter of ≈1 µm (Figure 2b). The FTIR spectra demonstrate the formation of dimeric carbazole (Figure 2c; Figure S3b, Supporting Information). For the PTO‐2CZ monomer, two vibratory peaks at 714 and 744 cm^−1^ are observed, which are induced by the disubstituted benzene ring in carbazole. While the polymer PPTO‐2CZ shows noticeably weaker peaks of disubstituted benzene rings and a newly generated yet dominant peak at 840 cm^−1^, which is attributed to the trisubstituted benzene ring, suggesting the synthesis of dimeric carbazoles.^[^ ^10b^ ^]^ These observations in FTIR spectra indicate the high coupling efficiency between carbazole groups. The XRD analysis indicates that the monomer PTO‐2CZ with good crystallinity (Figure 1c) gradually becomes amorphous when the PPTO‐2CZ is formed (Figure S4, Supporting Information), which also demonstrates the successful electropolymerization of PTO‐2CZ.

The influence of voltage on the electropolymerization and electrochemical performance was investigated by cycling the PTO‐2CZ cathodes in 1.5–4.0 and 1.5–4.5 V at 200 mA g^−1^, respectively. The galvanostatic charge–discharge (GCD) test in 1.5–4.0 V displays two typical redox plateaus of PTO at ≈3.10/2.95 and 2.58/2.32 V (Figure S5a, Supporting Information). In an extended voltage range of 1.5–4.5 V, the electrode is gradually stabilized by the electropolymerization of PTO‐2CZ above 4.0 V, and a high capacity was achieved due to the involvement of anion storage at high potential (Figure 2d; Figure S5b, Supporting Information). The dissolution behavior of the electrodes was further investigated by disassembling the batteries cycled for 100 cycles. The electrodes were immersed in 3 mL of the electrolyte for five days (Figure 2e). Similar UV–vis spectra with strong adsorption at 250–350 nm for the pristine electrode and that cycled between 1.5–4.0 V are obtained, accompanied by the pale red and dark red color of the solution, suggesting the strong dissolution of PTO‐2CZ. In comparison, the electrode cycled between 1.5–4.5 V showed no apparent absorption peak and the solution remained colorless. FTIR spectra further confirm the efficient depressing of dissolution. As shown in Figure S6 (Supporting Information), the FTIR spectra of the immersed solution are identical to that of the pure electrolyte, indicating poor solubility of PPTO‐2CZ. Benefiting from the reduced solubility of the in situ electropolymerized PTO‐2CZ cathode at high potential, the cycling stability at a cut‐off voltage of 4.5V is noticeably better than that at a cut‐off voltage of 4.0V. (Figure 2f). The morphology of the cycled electrodes within different voltage ranges was examined by SEM (Figure 2g–i). Using the pristine electrode as the reference, the active particles are apparently consumed with more conductive carbon nanotubes (CNTs) exposed to the surface after cycling between 1.5–4.0 V, demonstrating the severe dissolution of PTO‐2CZ (Figure 2g,h). In contrast, after cycling from 1.5–4.5 V, more active materials (PPTO‐2CZ) are sustained in the electrode as a result of the electropolymerization, which significantly depresses the dissolution during repeated charging/discharging(Figure 2i).

**Figure 3:**
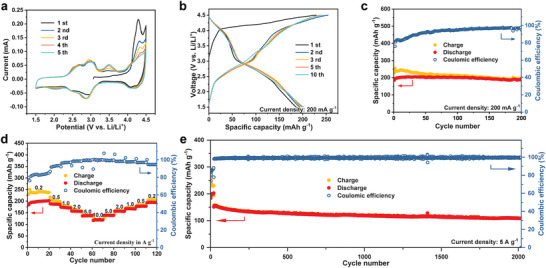
a) CV curves at 0.2 mV s^−1^ and b) galvanostatic charge/discharge profiles of the PTO‐2CZ cathode at 200 mA g^−1^. c) Cycling performance of PPTO‐2CZ cathodes at 200 mA g^−1^ in the voltage range of 1.5–4.5 V. d) Rate performance and of PPTO‐2CZ cathodes at different current densities. e) Long‐term cycling performance of the PPTO‐2CZ cathodes at 5 A g^−1^.

CV measurements were carried out at various cut‐off voltages from 3.9 to 4.5 V versus Li/Li^+^ at a scan rate of 1 mV s^−1^ to further investigate the electropolymerization in the PTO‐2CZ cathodes (Figure S7, Supporting Information). When charged to 3.9 and 4.1 V (Figure S7a,b, Supporting Information), only carbonyl groups of the PTO units react and two distinct redox couples at 3.24/2.76 and 2.71/2.19 V are displayed, indicating two pairs of two‐electron reaction of the four carbonyl groups, which is consistent with the typical isolation process of PTO‐based compounds. However, as the cut‐off voltage increased to 4.3 V (Figure S7c, Supporting Information), the two pairs of redox peaks between 1.5–3.5 V partially merged with the stronger peat centered at 2.88/2.78 V, indicating additional electrochemical reactions derived from the increased cut‐off voltage. For the cut‐off voltage of 4.5 V, a distinct anodic peak at ≈4.3 V and a new cathodic peak at a high voltage of 4.05 V in the first cycle are observed in addition to the typical redox couples of PTO (**Figure** **3a**; Figure S7d, Supporting Information), which can be attributed to the electropolymerization of PTO‐2CZ and the redox reaction of N atoms in carbazole units. In the following five cycles, the anodic peak gets weakened and stable, indicating the accomplishment of the electropolymerization. As shown in Figure 3b, the galvanostatic charge/discharge profiles of the PTO‐2CZ cathode exhibit a long flat charge voltage plateau around 4.3 V during the first charge process, which is consistent with the electropolymerization peak and the redox peak of N atoms in CV measurements. The initial high charge capacity of PTO‐2CZ can be ascribed to the electropolymerization under high voltage, as confirmed by three‐electrode tests. Furthermore, the oxidation of the carbazole unit between 4.0–4.5 V during charging could induce anion insertion and contribute additional capacity, which is demonstrated in the mechanism study via ex situ characterizations in the following section. Thereafter, two sloping voltage plateaus are presented at 2.87 and 4.15 V in the subsequent cycles with good reversibility. The discharge capacity for the first cycle is 202 mA h g^−1^. Notably, the conductive additive (Ketjen Black) exhibits a capacity of 62 mAh g^−1^ at 0.2 A g^−1^ when pure Ketjen Black was applied as the active cathode (Figure S8, Supporting Information). The cycling performance of the PPTO‐2CZ cathode was further evaluated at different cut‐off voltages from 4.3 to 4.7 V at a current density of 500 mA g^−1^ (Figure S9, Supporting Information). As the cut‐off voltage increased from 4.3 to 4.7 V, the discharge capacity increased from 185 to 244 mA h g^−1^. It is noteworthy that the low‐voltage plateau corresponding to the *n*‐type reaction did not significantly change when the charge cut‐off voltage varied. However, the capacity contribution from the *p*‐type reaction gradually increased, indicating that a more complete *p*‐type reaction occurs at a higher cut‐off voltage. However, the Coulombic efficiency gradually decreased with the increase of cut‐off voltage, and the cycling stability at 4.7 V was worse than that at 4.5 V, which can be ascribed to the partial decomposition of the electrolyte at high voltage (Figure S10, Supporting Information).

Figure 3c shows the stable cycling of PPTO‐2CZ between 1.5–4.5 V, where a high capacity of 196 mAh g^−1^ is sustained after 200 cycles at a current density of 200 mA g^−1^, corresponding to 97% capacity retention. It's noteworthy that the initial Coulombic efficiency of the battery is relatively low; however, it exhibits a progressive increase through successive cycles, eventually reaching a state of stability after ≈50 cycles. This can be ascribed to the continuous electropolymerization at low current density before the termination, which would provide additional irreversible charge capacity. The specific capacity slightly decreases from 202 to 119 mA h g^−1^ when the current density increases from 0.2 to 10 A g^−1^, and it recovers to 195 mA h g^−1^ when the current density returns to 0.2 A g^−1^ (Figure 3d). Significantly, the bipolar characteristic of PPTO‐2CZ is fully consistent with the two plateaus that are clearly observed at all current densities (Figure S11, Supporting Information). The long‐term cycling of the PPTO‐2CZ cathodes was also conducted at a high current density of 5 A g^−1^ (Figure 3e). After the activation processes at 200 mA g^−1^ for 20 cycles, the capacity gradually decreased from 150 to 109 mA h g^−1^ after 2000 cycles, corresponding to a retention of 72.7%. The Coulombic efficiency is rapidly promoted to above 99% after activation. The excellent rate performance and cycling stability can be attributed to the enhanced conjugation degree resulting from the D–A structural of PPTO‐2CZ as it induces an electron “push‐pull” effect along the polymer chains.

**Figure 4:**
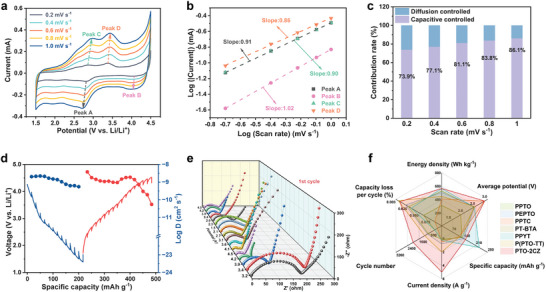
a) CV curves of PPTO‐2CZ cathodes at different scan rates from 0.2 to 1.0 mV s^−1^. b) The calculated b values. c) The ratio of pseudocapacitive‐ and diffusion‐controlled charge storage contributions at different scan rates from 0.1 to 1.0 mV s^−1^. d) GITT curves and the calculated diffusion coefficients of charge/discharge processes for PPTO‐2CZ cathodes. e) In situ EIS profiles of PTO‐2CZ cathode in the first cycle. f) Radar chart of various factors for PPTO‐2CZ cathode with a comparison with the literature.

The reaction process was further investigated by CV measurements at various voltage scan rates from 0.2 to 1.0 mV s^−1^ (**Figure** **4**a). A faradaic and a capacitive process typically contribute to the total capacity. The relationship between peak current (i) and voltage scan rate (*v*) can be studied using the formula *i* = *av^b^*, where a and b are parameters. A capacitive process has a b value close to 1.0, whereas a faradaic process has one close to 0.5. The b values of the electropolymerized PTO‐2CZ cathodes in peaks A, B, C, and D are 0.91, 1.02, 0.90, and 0.86 respectively, indicating the capacity of the PPTO‐2CZ cathodes are mainly controlled by a capacitive contribution (Figure 4b). Furthermore, the contribution of the capacitive component at a specific scan rate is also calculated using the equation

(1) $$i\left(V\right)=k_{1}v+k_{2}v^{1/2}$$

where *k*_1_ *v* stands for the pseudocapacitive contribution and *k*_2_ *v* ^1/2^ for the diffusion‐controlled contribution. At a scan rate of 1 mV s^−1^, 86.1% pseudocapacitive contribution can be achieved, which affords the outstanding rate performance at high current densities (Figure 4c; Figure S12, Supporting Information). Furthermore, the ion diffusion coefficients (D) of the PPTO‐2CZ cathode were estimated using the galvanostatic intermittent titration technique (GITT) method after 20 cycles at 200 mA g^−1^ (Figure 4d). The lithium‐ion diffusion coefficients (*D*_Li_^+^) of the PPTO‐2CZ cathode is calculated to be 1.39 × 10^−10^ – 5.59 × 10^−10^ cm^2^ s^−1^ when discharging and 5.55 × 10^−11^ – 3.81 × 10^−9^ cm^2^ s^−1^ when recharging as illustrated in Figure 4d, which suggests the stable and efficient ionic conductivity of the PPTO‐2CZ. In addition, the diffusion coefficient steadily decreases during the charging process to 4.5 V, and the lower coefficient at high voltage is attributed to the high intercalation barrier of the substantial volume of PF^6−^ anions. The intercalated anions are helpful for improving the Li^+^ kinetic by the electrostatic interaction, thus a high coefficient is obtained at the beginning of the discharging process. This phenomenon fits well with the dual‐ion storage mechanism.^[^ ^10c^ ^]^ In situ electrochemical impedance spectroscopy (EIS) was also employed to study the real‐time evolution of interface properties of PPTO‐2CZ cathodes (Figure 4e; Figure S13, Supporting Information). The Nyquist plots clearly show that the battery exhibits low charge transfer resistance (R_ct_) at high potentials, suggesting that PPTO‐2CZ has rapid kinetics at high potentials, which aligns with the GITT result. In the initial cycle (Figure 4e), the cathode exhibits an additional semicircle during the electropolymerization process, which is related to the formation of PPTO‐2CZ and the cathode electrolyte interphase. Moreover, the cathode shows an overall lower R_ct_ after cycling, implying that the in situ electropolymerization leads to remarkably improved kinetics.

In addition to remarkable cycling stability at a high current density of 5 A g^−1^ (Figure 2e), the energy density of the PPTO‐2CZ cathode is determined to be 554 Wh kg^−1^, which is comparable to that of commercial inorganic cathode materials and outperforms many previous reported PTO‐based material cathodes (Table S1, Supporting Information).^[^ ^8^, ^14^ ^]^ Compared to the recently reported D‐A electrodes and bipolar‐type cathodes for lithium‐ion batteries, PPTO‐2CZ‐based batteries also show competitive electrochemical performance (Tables S2 and S3, Supporting Information).^[^ ^4^, ^5^, ^9^, ^15^ ^]^

**Figure 5:**
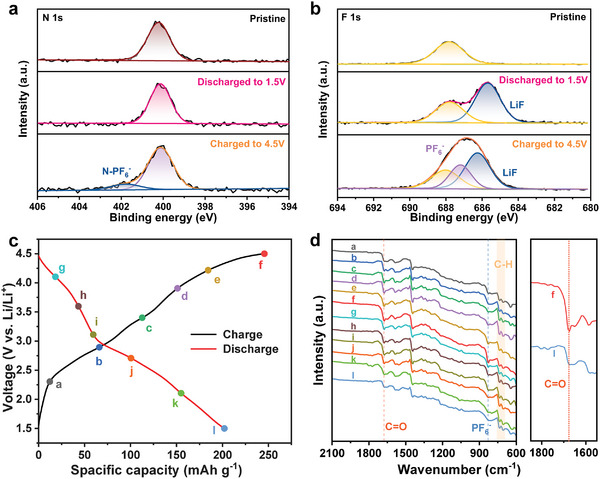
High‐resolution a) N 1s and b) F 1s spectra of the PPTO‐2CZ cathodes at different charge/discharge states. c) Galvanostatic charge/discharge profiles and d) FTIR spectra at different charge/discharge states marked in (c).

Ex situ X‐ray photoelectron spectroscopy (XPS) and FTIR spectroscopy on the electrodes at various charge/discharge states were used to investigate the charge storage of the PPTO‐2CZ cathode (**Figure** **5**). The full survey XPS spectrum of the pristine PTO‐2CZ cathode shows three peaks located at 285, 400, and 687 eV, corresponding to C 1s, N 1s, and F 1s, respectively (Figure S14a, Supporting Information). Noticeably, the poly (vinylidene fluoride) (PVDF) binder is responsible for the peak associated with fluorine in the XPS spectrum of the pristine PTO‐2CZ cathode. After being charged to 4.5 V, two new peaks appeared at 136 and 193 eV, corresponding to P 2p and P 2s respectively, accompanied by the increase of the fluorine content, indicating the insertion of the PF_6_^−^ anions. On the contrary, the intensities of the P and F peaks became lower and the contents decreased accordingly after being discharged to 1.5 V, suggesting reversible extraction of PF_6_^−^ anions. Additionally, a novel peak known as Li 1s was observed at 56 eV, demonstrating an *n*‐type reaction of the lithium‐ion intercalated into the PTO units of the PPTO‐2CZ cathode (Figure S14, Supporting Information). High‐resolution F 1s and N 1s XPS spectra provide a clearer view of the reversible process (Figure 5a,b). A new N peak at 402.5 eV, which is attributed to the N‐PF_6_^−^ interaction, appeared along with the strong 686.7 eV signal associated with PF_6_^−^ anions at the charged state (4.5 V). When discharged to 1.5 V, these signals disappeared, demonstrating that the N atoms of PPTO‐2CZ can efficiently extract/release anions, resulting in good reversibility. In the C 1s spectra (Figure S14c, Supporting Information), the content of the C═O peak decreases with the appearance of C─O peak, showing an *n*‐type reaction during the discharge process. The XPS spectra of O 1s also exhibit a commensurate peak shift at 532.2eV (Figure S14d, Supporting Information). At the discharged state (1.5 V), a clear observation of the peak shifting toward lower energy by 1.0 eV is observed. The origin of energy transfer can be attributed to the increased electron density around oxygen atoms during the discharge process, consistent with the formation of C─O─Li bonds. Moreover, ex situ FTIR spectra were collected at various charging and discharging states marked on the voltage profiles in Figure 5c. It is evident that a strong anionic PF_6_^−^ peak at 836 cm^−1^ is clearly obtained at 4.5 V (Figure 5d), indicating the interaction of PF_6_^−^ with PPTO‐2CZ molecules, and the peak becomes weaker during discharge and disappears at 1.5 V, pointing to a reversible electrostatic interaction between N atoms and PF_6_^−^ anions. Similarly, the carbonyl group peaks at 1682.6 cm^−1^ exhibit reversible variation during the charge and discharge process, which is indicative of an *n*‐type carbonyl group reaction. These results are well consistent with the XPS analysis, demonstrating the bipolar feature of PPTO‐2CZ cathodes during electrochemical reactions.

**Figure 6:**
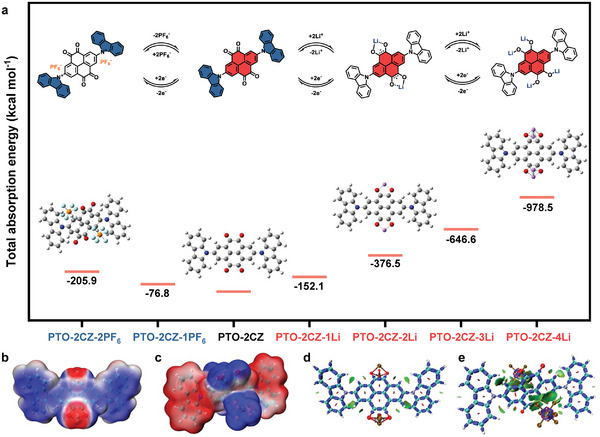
a) Spatial structure and total absorption energy between PTO‐2CZ‐4Li^+^ and PTO‐2CZ‐2PF_6_^−^. b,c) The electron density difference isosurfaces of PTO‐2CZ‐4Li^+^ and PTO‐2CZ‐2PF_6_^−^. The blue and red isosurfaces represent the region in which the electron density increases and decreases after the coordination of 4Li^+^ and 2PF_6_^−^ respectively. d,e) The interaction region indicator (IRI) analysis of PTO‐2CZ‐4Li^+^ and PTO‐2CZ‐2PF_6_^−^.

Theoretical calculations based on density functional theory (DFT) have been performed to identify the sequential structural and corresponding energy evolution between PTO‐2CZ‐4Li and PTO‐2CZ‐2PF_6_. The adsorption of PTO‐2CZ at different states and total adsorption energy were carefully simulated (**Figure 6**a; Figure S15, Supporting Information). It's shown that cations (Li^+^) and anions (PF_6_^−^) have distinctive interactions with bipolar PTO‐2CZ in both the discharged and charged states. Specifically, Li^+^ ions directly interact with the oxygen atoms of PTO‐2CZ, resulting in absorption energies of PTO‐2CZ with one, two, three, and four Li^+^ ions of −152.1, −188.2, −215.5, and −244.6 kcal mol^−1^, respectively. On the other hand, PF^6−^ ions are located in proximity to nitrogen atoms of the carbazole units. The average absorption energies of PTO‐2CZ with one and two PF_6_^−^ ions are −76.8 and −102.9 kcal mol^−1^, respectively. Notably, the interaction with PF_6_^−^ ions in the charged state leads to a significant distortion of the PTO‐2CZ structure, owing to the large ionic size of PF_6_^−^ ions. The PTO‐2CZ molecule has the ability to attract four Li^+^ ions and two PF_6_^−^ ions, which provides a significant theoretical capacity with respect to the transfer of six electrons. Furthermore, PTO‐2CZ displays a narrow energy gap of 2.01 eV and an extended highest occupied molecular orbital (HOMO), which could be attributed to the high conjugation degree resulting from the D‐A structure of PTO‐2CZ (Figure S16, Supporting Information). Subsequently, in this study, electron density difference isosurfaces (as displayed in Figure 6b,c) were examined, and natural population analysis (NPA) of PTO‐2CZ‐4Li and PTO‐2CZ‐2PF_6_ (Table S4, Supporting Information) was conducted. The well‐distributed charge density observed with Li^+^ and PF_6_^−^ during discharge and charge operations suggests excellent cycling stability. These results suggest that an enolization reaction occurred in PTO units during the formation of PTO‐2CZ‐4Li, while relatively weak chemical bonds of N─PF_6_^−^ are observed during the formation of PTO‐2CZ‐2PF_6_. Additionally, the interaction region indicator (IRI) analysis was performed to estimate intermolecular interaction displayed in Figure 6d,e. The green region represents favorable dipole interactions between PF_6_^−^ and the PTO‐2CZ complex, which is consistent with the electron density difference and NPA. The findings are also consistent with the ex situ FTIR and XPS analysis, dictating the importance of dipole interactions for the high capacity, excellent rate capability, and stable long‐term cycling of the PPTO‐2CZ cathode.

## 3 Conclusion

In conclusion, a novel D‐A bipolar cathode material was constructed by in situ electropolymerization using PTO‐2CZ containing PTO *n*‐type units and carbazole p‐type units. The polymerized PTO‐2CZ can significantly depress the severe dissolution of small molecules and greatly improve the cycling stability. It's proved that the electropolymerized PTO‐2CZ cathode possesses good reaction kinetics and excellent electrochemical performance, demonstrating a high reversible capacity of 202 mA h g^−1^ at 200 mA g^−1^, high discharge voltage of 2.87 and 4.15 V, a high energy density of 554 Wh kg^−1^ with outstanding rate performance of 119 mA h g^−1^ at 10 A g^−1^, and increased cycling stability over 2000 cycles with a capacity retention of 72.7% at 5 A g^−1^. Ex situ XPS and FTIR spectroscopy were employed to investigate the reaction mechanism of PPTO‐2CZ, which revealed that it included a *p*‐type reaction of N atoms in the carbazole units with anions and an *n*‐type reaction of carbonyls in PTO units with cations. Our research offers an efficient approach to mitigate the dissolution problem and sheds light on the rational design of organic electrode materials with high energy density for lithium‐ion batteries.

## 4 Experimental Section

### Synthesis of PTO‐2CZ

PTO‐2CZ was synthesized using carbazole and compound 1 via a simply modified Ullmann condensation method (Schemes S1 and S2, Supporting Information). In a Schlenk tube, compounds 1 (596 mg, 1 mmol, 1.0 eq.), carbazole (502 mg, 3 mmol, 3.0 eq.), K_2_CO_3_ (829 mg, 6 mmol, 6.0 eq.), catalyst CuI (114 mg, 0.6 mmol, 0.6 eq.), and ligand L‐proline (69 mg, 0.6 mmol, 0.6 eq.) were added. These compounds were dissolved into dry DMF (25 mL). After bubbling with argon for 20 min and ultrasound for 5 min, the tube was sealed and the reaction proceeded with stirring and refluxing at 160 °C for 72 h. After the system cooled down to room temperature, The solvent was removed from the crude product by vacuum distillation method and then dichloromethane was added to dissolve the formed precipitate. After that, the crude product was purified by silica column, eluting with a mixture of dichloromethane: petroleum ether (1:2) and then purified by recrystallization in dichloromethane to give a white solid as protected intermediate, p‐PTO‐2CZ (400 mg, yield 52%). ^1^H NMR (300 MHz, CDCl_3_, δ): 8.17 (d, 4H), 8.07 (s, 4H), 7.54 (d, 4H), 7.47 (t, 4H), 7.32 (t, 4H), 4.24 (br, 8H), 3.80 (br, 8H). After that, the obtained product was added into a mixture of trifluoroacetic acid (TFA)‐H_2_O (9:1, 40 mL) and refluxed in a sealed tube under argon protection for 24 h. The system was then cooled down to room temperature and poured into cold water, after filtration, the filtrate was washed with water and EtOH to yield bluish violet powder as PTO‐2CZ (278 mg, yield 90%). ^1^H NMR (300 MHz, DMF‐d7, δ): 8.69 (s, 4H), 8.39 (d, 4H), 7.64 (d, 4H), 7.60 (t, 4H), 7.44 (t, 4H).

### Coin‐Type Cell Fabrication and Electrochemical Measurements

PTO‐2CZ electrodes were fabricated by 50 wt.% PTO‐2CZ powder, 40 wt.% Ketjen Black (KB) and 10 wt.% poly(vinylidene fluoride) (PVDF) binder. The obtained slurry was coated on carbon‐coating Al foil by a doctor blade and then dried at 80 °C in the vacuum oven for over 8 h. The loading of active materials is ≈1.0 mg cm^−2^. Coin cell was assembled by the counter electrodes of lithium metal, polypropylene separators (Celgard 2400, LLC Corp., USA), and electrolytes of 1 m LiPF_6_ in ethylene carbonate: ethyl methyl carbonate (EC: EMC) (3:7 by volume). The galvanostatic charge/discharge performance was performed on the Land test system (CT2001A, China). Cyclic voltammograms at different scan rates between 1.5–4.5 V and Electrochemical Impedance Spectroscopy (EIS) spectra in the frequency of 1 000 000–0.01 Hz at 5 mV amplitude were recorded using Solartron 1470 Electrochemical Interface (Solartron Metrology, UK).

### Ex Situ Characterization of the Electrodes

The electrodes for the ex situ FTIR test and XPS test were prepared by grinding PTO‐2CZ, carbon nanotubes (CNTs), and polymer binders (polyvinylidene fluoride) (PVDF) in a weight ratio of 7:2:1. The electrodes for the SEM images at different cut‐off voltages were fabricated by 30 wt.% PTO‐2CZ powder, 50 wt.% CNTs, and 20 wt.% PVDF binder.

### Three‐Electrode Experiments

CH Instruments 660 C electrochemical workstation was carried out to test the electrochemical property of the PTO‐2CZ electrode. This experiment was applied in the conventional three‐electrode system. A glassy carbon electrode of 2 mm dia. and indium‐tin‐oxide (ITO) conductive film glass was used as the working electrode, Pt wire served as the counter electrode, whereas the silver wire electrode was used as a reference electrode. Additionally, in each experiment, the reference electrode potential was monitored using ferrocene as a standard. Cyclic voltammetry measurements in the solutions using glassy carbon and ITO conductive film as working electrodes were carried out at the scan rate of 100 and 50 mV s^−1^ at room temperature that was bubbled with argon for 10 min before each experiment. A typical sample concentration was 0.3 mm in the dried dichloromethane solution containing 0.1 m tetrabutylammonium hexafluorophosphate (Bu_4_NPF_6_, 98%, Sigma) as the supporting electrolyte.

### Galvanostatic Intermittent Titration Technique (GITT)

The diffusion coefficient (*D* ^GITT^) can be calculated by the GITT method according to the following equation:

(2) $$D^{GITT}=\frac{4}{\pi \tau }\;{\left(\frac{m_{B}V_{m}}{M_{B}S}\right)}^{2}{\left(\frac{\Delta E_{S}}{\Delta E_{\tau }}\right)}^{2}$$

where τ is the duration of the current pulse (1200 s); m_B_ and M_B_ are mass and molar mass of PTO‐2CZ (0.000 895 g, 592.61g mol^−1^), respectively; *V*_m_ is molar volume of PTO‐2CZ (748 cm^3^ mol^−1^), S represents the electrode‐electrolyte interface area, which is replaced by the geometric area of the electrode, 1.13 cm^2^); Δ*E*s is the steady‐state potential change (*V*) between two adjacent relaxations of 18000 s; Δ*E*τ is the potential change (*V*) during a current pulse at 15 µA after being eliminated the iR drop. ∆*E*s is the potential change between neighboring relaxation end time; ∆*E*τ is the potential change caused by every constant current charge/discharge process.

## Conflict of Interest

The authors declare no conflict of interest.

## Supporting information

Supporting Information

## Data Availability

The data that support the findings of this study are available from the corresponding author upon reasonable request.
