# Supplementary material for: Electropolymerization of Donor–Acceptor Conjugated Polymer for Efficient Dual‐Ion Storage
Source: Adv Sci (Weinh). 2024 Apr 6;11(23):2310239. doi: 10.1002/advs.202310239 (PMC11187866; doi:10.1002/advs.202310239)
Supplement: Supplementary file 1 — Supporting Information [file ADVS-11-2310239-s001.pdf]

ADVANCED SCIENCE
Open Access

# Supporting Information

for *Adv. Sci.*, DOI 10.1002/advs.202310239

Electropolymerization of Donor–Acceptor Conjugated Polymer for Efficient Dual-Ion Storage

*Xianhe Chen, Weisheng Zhang, Chenxing Zhang, Yuxuan Guo, Ao Yu, Shilin Mei**
*and Chang-Jiang Yao**

# Supporting Information

## Electropolymerization of donor-acceptor conjugated polymer for efficient dual-ion storage

*Xian-He Chen, Wei-Sheng Zhang, Chen-Xing Zhang, Yu-Xuan Guo, Ao Yu, Shi-Lin Mei,* Chang-Jiang Yao**

## Contents:

**1. Experimental Procedures........................................................................S1**

**2. Supplementary Data..............................................................................S4**

**3. References…………..…………………………………………………..……...S18**

### 1. Experimental Procedures

**Materials and Characterization**

All starting chemicals and solvents were obtained from Energy Chemical or MREDA, and used without further purification. Nuclear magnetic resonances ($^1$H NMR) were conducted on Bruker Avance III 400MHz. Solid-state $^{13}$C NMR spectra were recorded using a Bruker NMR spectrometer. Transform Infrared Spectroscopy (FTIR) was recorded with Bruker 46 TENSOR Π. Thermal gravimetric analysis (TGA) was carried out with Hitachi 7300 at a heating rate of 10 °C min$^{-1}$ under nitrogen atmosphere. The microstructure and morphology were observed by high-resolution cold field-emission scanning electron microscopy (SEM, Regulus 8230) equipped with energy dispersive spectroscopy (EDS) for elemental analysis. X-ray diffraction (XRD) patterns were

collected on a Rigaku Ultima IV using Cu Kα radiation (λ= 1.5406 Å, 40 kV and 40 mA). Ultraviolet-visible spectroscopy (UV-vis, Shimadzu UV-3600 plus, Japan) and X-ray photoelectron spectroscopy (XPS, Thermo Scientific K-Alpha, USA) was applied to analyze the chemical composition of the pristine and cycled electrode. The cycled electrodes used for the characterization were washed with Dimethyl carbonate (DMC) and dried.

**Synthesis of PTO-2Br and compound 1**

As shown in Scheme S1, PTO-2Br was synthesized according to literatures,[1, 2] and light-yellow powder was obtained with the yield of 87%. $^1$H NMR (300 MHz, $CDCl_3$, δ): 8.60 (s, 4H, Ar H).

To 20 mL ethylene glycol and 20 mL toluene were added PTO-2Br (844 mg, 2 mmol) and $TsOH.H_2O$ (760 mg, 4 mmol). The mixture was heated at 140 °C for 36 h under argon protection. After the solution cooled down to room temperature, the product was purified by filtration and washing with water and EtOH to yield 937 mg of compound **1**, as a white powder in 80.5% yield. $^1$H NMR (300 MHz, $CDCl_3$, δ): 7.89 (s, 4H), 4.21 (s, 8H), 3.67 (s, 8H).

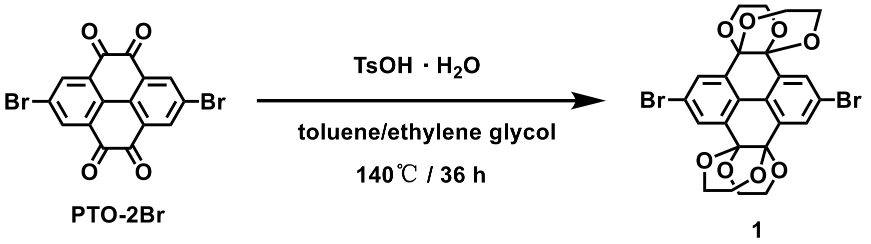


**Scheme S1.** Synthesize routes of compound **1**.

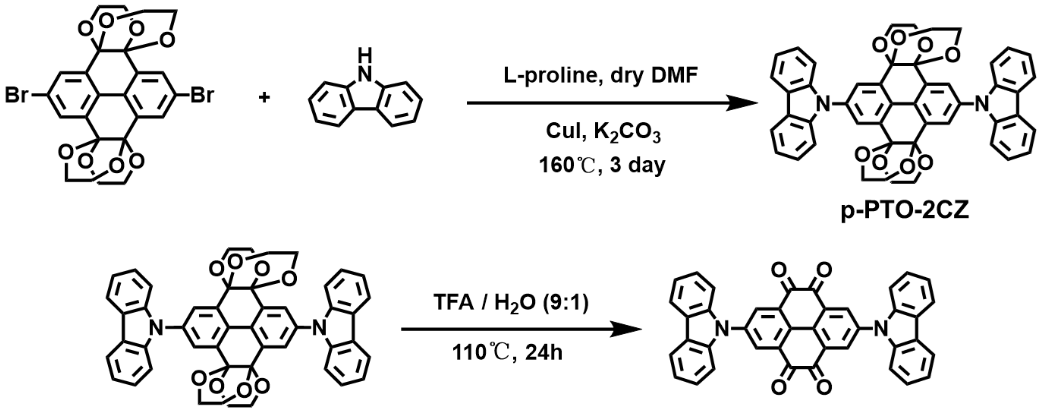


**Scheme S2**. Synthetic route of PTO-2CZ by Ullmann reaction.

## Computational methods

All calculations were performed with Gaussian 16 program[3]. Structures were optimized under B3LYP-6-31G(d,p)* and frequency calculations were carried out under the same level to confirm the stationary points as minima (no imaginary frequencies). Free energies (in kcal $mol^{-1}$) obtained were discussed in the manuscript. The electron density difference analysis and the interaction region indicator (IRI) analysis[4] of PTO-2CZ-4Li and PTO-2CZ-4$PF_6$ were carried out with the help of Multiwfn program[5] and the results were visualized by the VMD program[6].

## 2. Supplementary Data

a

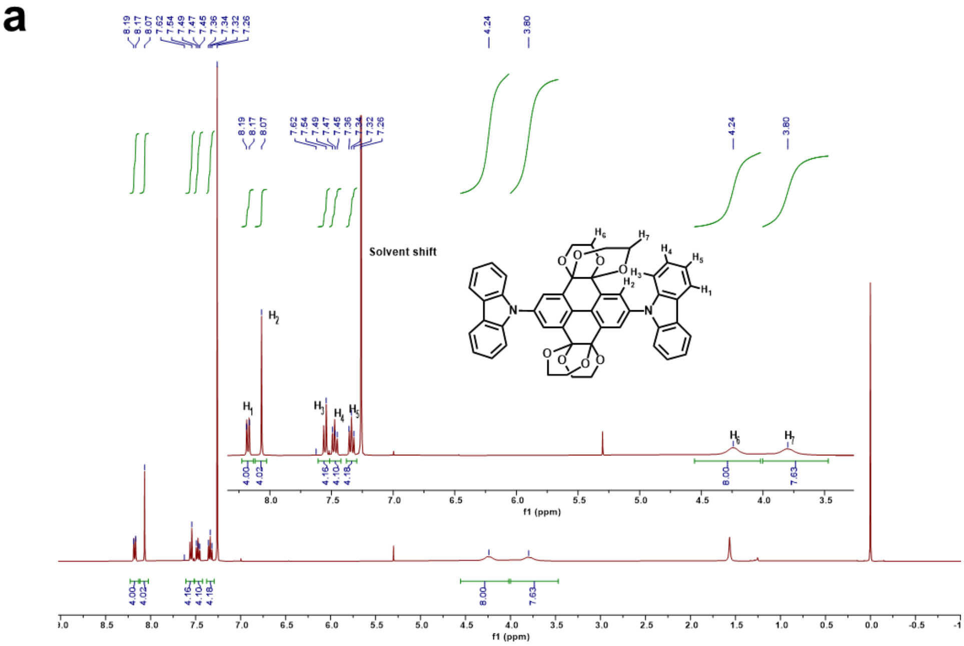


b

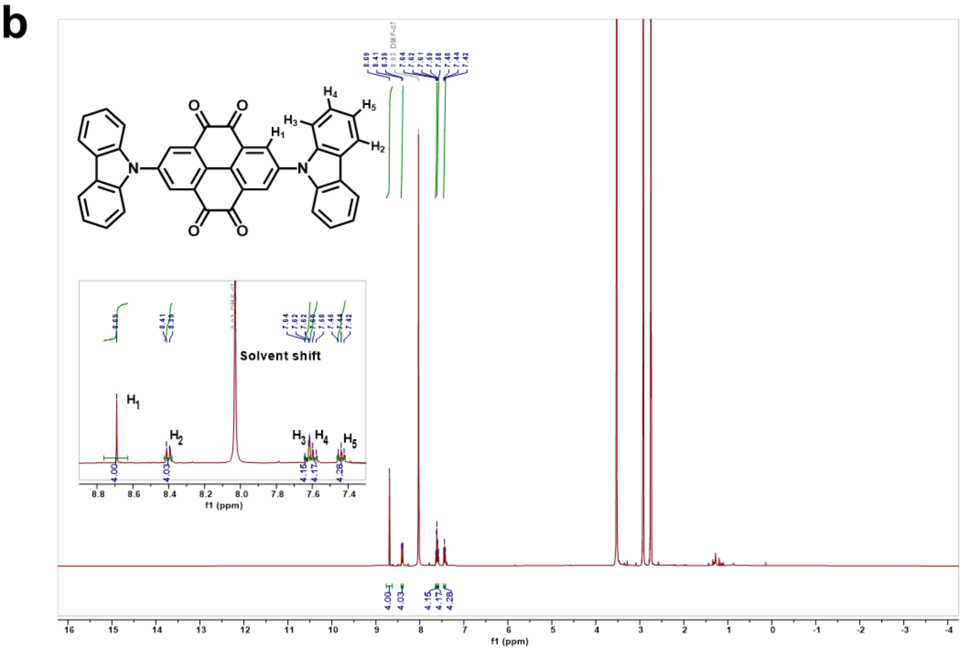


**Figure S1.** (a) $^{1}$H NMR spectra ($CDCl_3$, 300 MHz) of p-PTO-2CZ and (b) $^{1}$H NMR spectra (DMF-d7, 300 MHz) of PTO-2CZ.

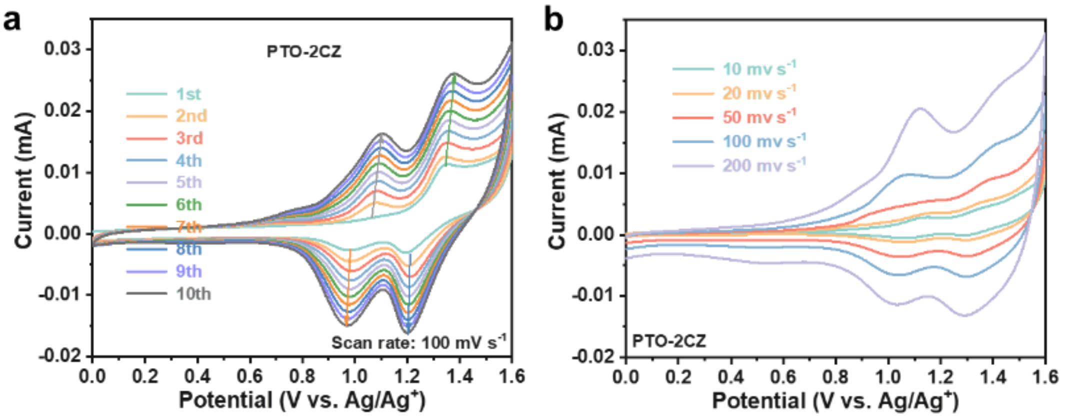


**Figure S2.** (a) The first to tenth cycle of CV of 3mM PTO-2CZ and 1M Tetrabutylammonium hexafluorophosphate in dry $CH_2Cl_2$ with glassy carbon as working electrode, Pt wire as counter electrode and Ag/Ag$^{+}$ as reference electrode; (b) CV curves of polymer PTO-2CZ film in monomer-free electrolyte under different scan rates.

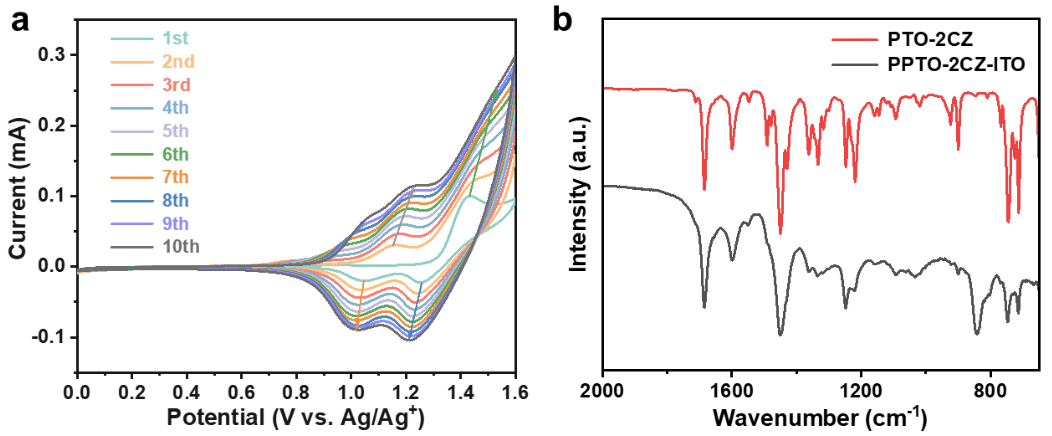


**Figure S3.** (a) The first to tenth cycles of CV of 3mM PTO-2CZ in dry dichloromethane ($CH_2Cl_2$) with ITO-coated glass as working electrode, Pt wire as counter electrode and Ag/Ag$^{+}$ as reference electrode; (b) FTIR spectrum of polymer PTO-2CZ film.

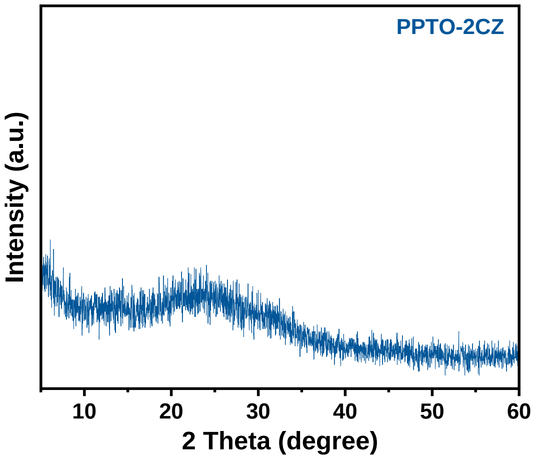


**Figure S4.** XRD pattern of PPTO-2CZ.

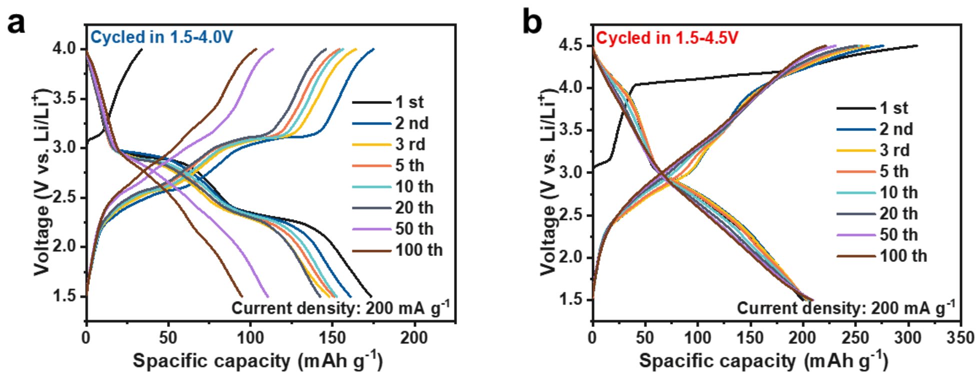


**Figure S5.** Galvanostatic charge/discharge profiles of PTO-2CZ cathodes at different voltage ranges (a) 1.5-4.0 V and (b) 1.5-4.5 V.

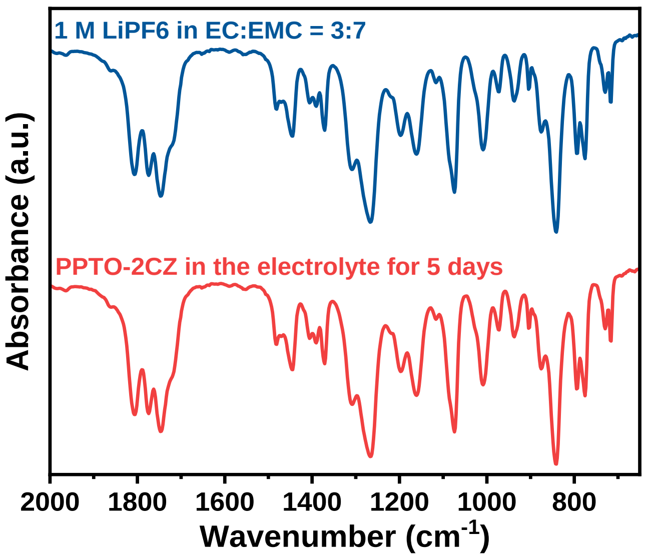


**Figure S6.** FTIR of the electrolytes immersed by PPTO-2CZ for 5 days.

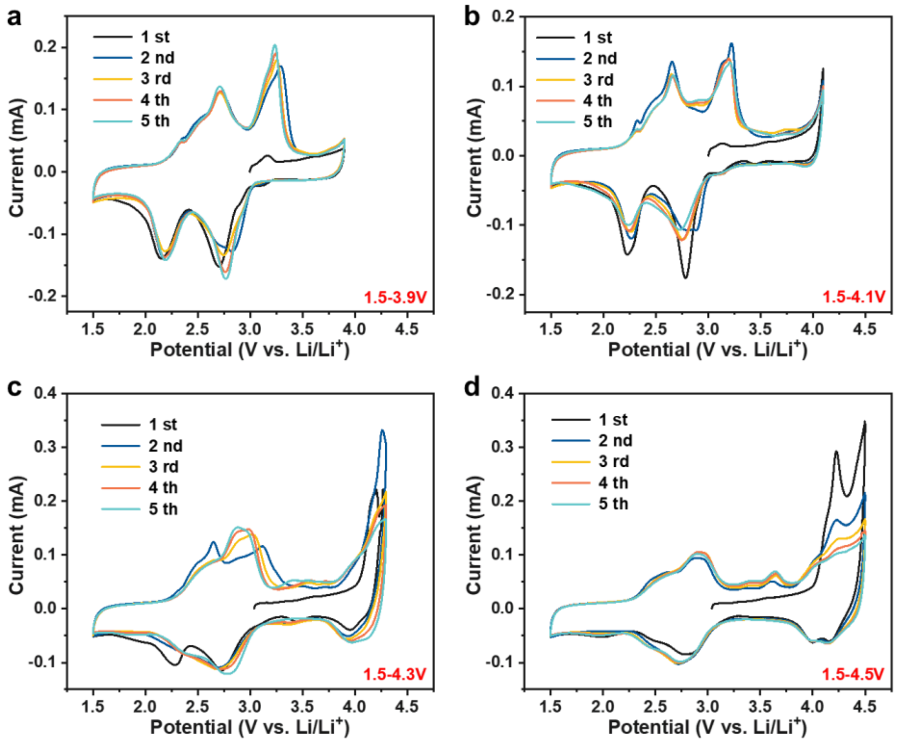


**Figure S7.** CV curves of PTO-2CZ cathodes at different voltage ranges of (a) 1.5-3.9 V, (b) 1.5-4.1 V, (c) 1.5-4.3 V, and (d) 1.5-4.5 V vs. $Li/Li^+$ at a scan rate of 1.0 mV $s^{-1}$ in coin cells.

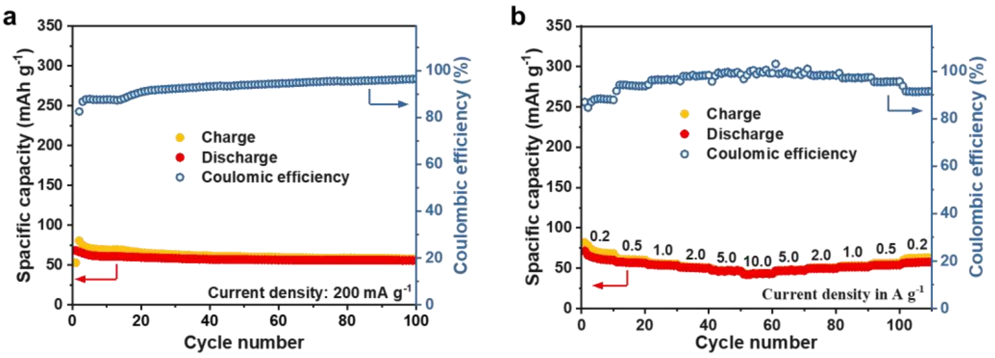


**Figure S8.** (a) Cycling performance of Ketjen Black electrodes at 200 mA $g^{-1}$ in 1.5-4.5 V. (b) Rate performance of Ketjen Black electrodes at different current densities.

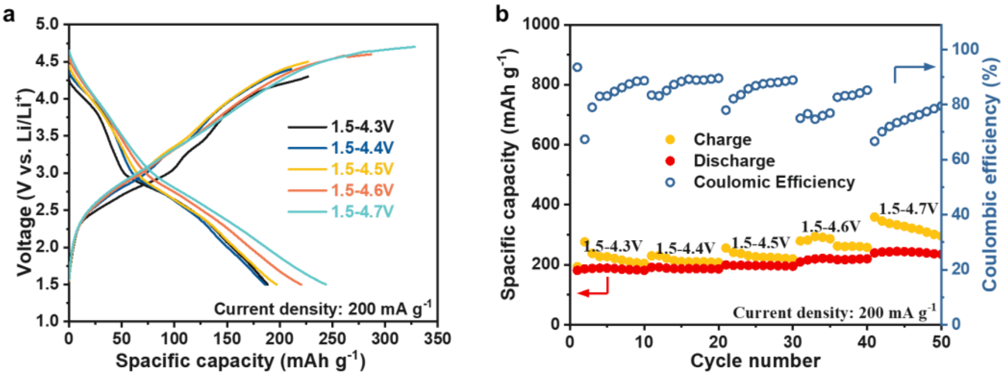


**Figure S9.** (a) Charge/discharge profiles and (b) electrochemical performance of PTO-2CZ cathodes at various high cutoff voltages from 4.3 V to 4.7 V at a current density of 500 mA h g-1.

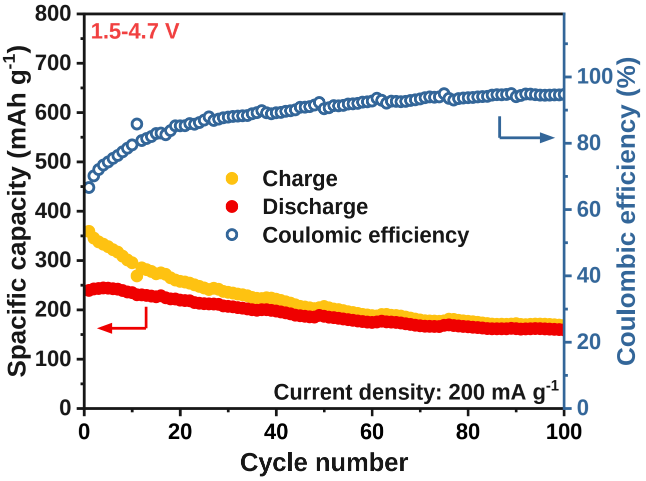


**Figure S10.** Cycling performance of PTO-2CZ electrodes at 200 mA g-1 in 1.5-4.7 V.

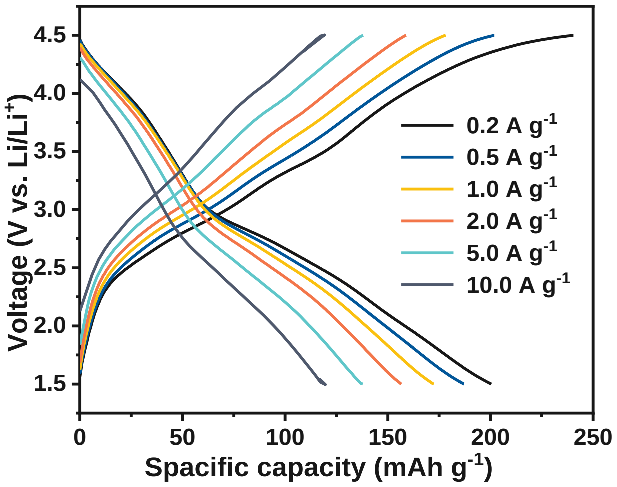


**Figure S11.** Galvanostatic charge/discharge profiles of PTO-2CZ cathodes at different current densities in the voltage range of 1.5-4.5 V.

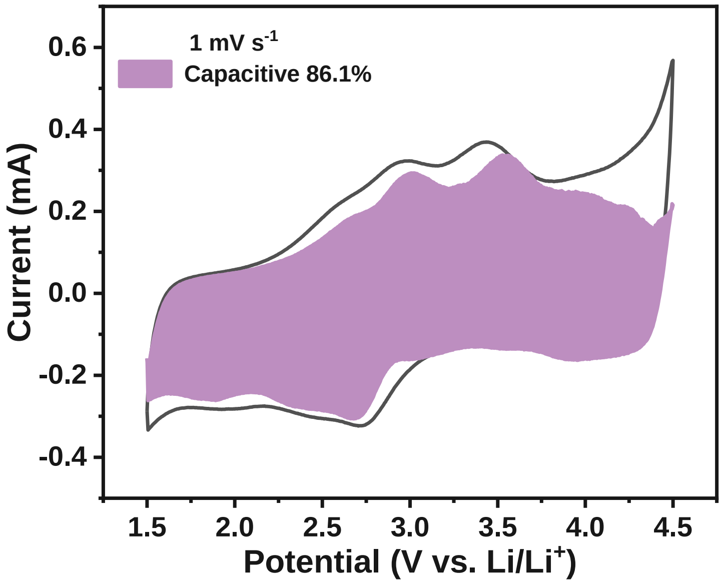


**Figure S12.** Capacitive at a scan rate of 1 mV s$^{-1}$.

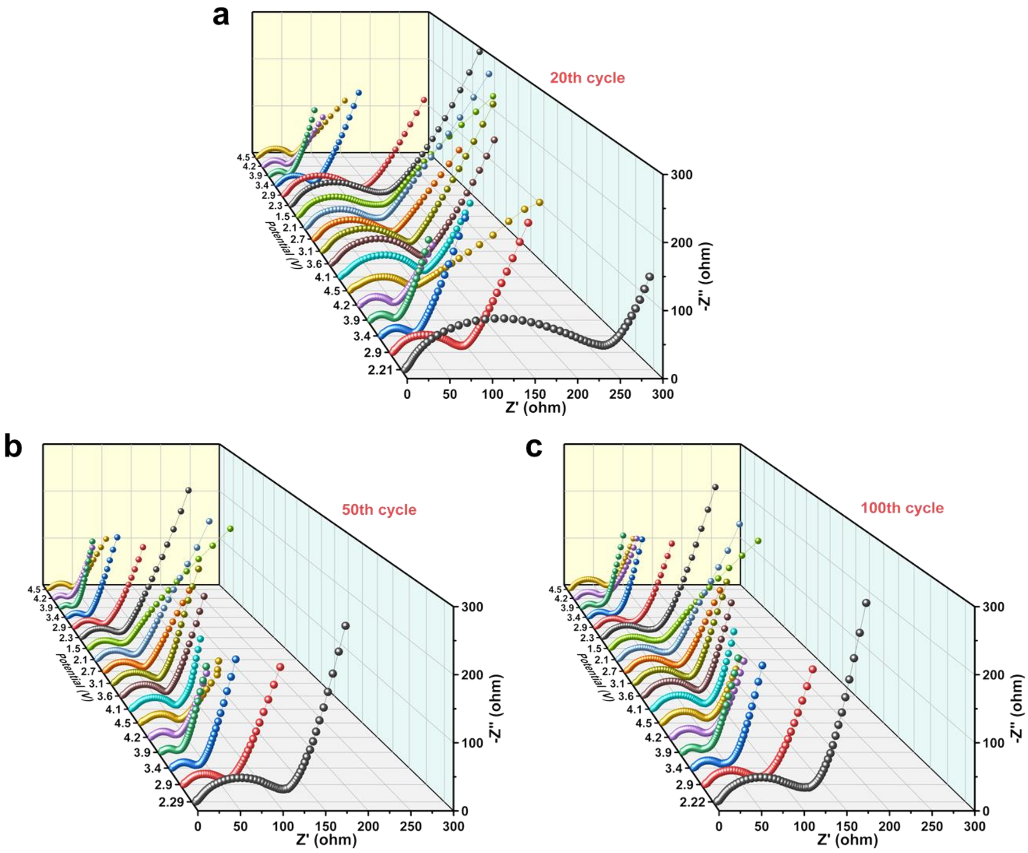


**Figure S13.** *In-situ* EIS profiles of PTO-2CZ cathode at (a) the 20th, (b) the 50th, (c) the 100th cycles.

**Table S1.** A comparison table includes several PTO-based electrodes for lithium-ion batteries.

| Organic cathode | Range of Potential | Reversible capacity (mA h $g^{-1}$@A $g^{-1}$) | Cycle life | Rate capacity (mAh $g^{-1}$@A $g^{-1}$) | Ref |
|---|---|---|---|---|---|
| PT-2$NO_2$ | 1.0-3.5 V | 250.4@0.05 | 82.3 mA h $g^{-1}$ at 0.5 A $g^{-1}$ (500 cycles) | 101.3@1.0 | [1] |
| PPTO | 1.5-3.5 V | 234@0.02 | 74.0 % at 0.1 A $g^{-1}$ (953 cycles) | 22@0.3 | [2] |
| PEPTO | 1.5-3.5 V | 244@0.02 | 79.0 % at 0.8 A $g^{-1}$ (1000 cycles) | 98@1.5 | [2] |
| PTO-$NH_2$ | 1.5-3.8 V | 350@0.05 | 83% at 0.05 A $g^{-1}$(100 cycles) | 292@1.0 | [7] |
| PPTC | 1.5-3.5 V | 142.5@0.05 | 74.6% at 0.5 A $g^{-1}$ (300 cycles) | 120.6@1.0 | [8] |
| PPTODB | 1.5-3.5 V | 198@0.02 | 68.3 % at 0.02 A $g^{-1}$ (150 cycles) | 100@1.0 | [9] |
| PT-BTA | 1-3.5 V | 166.7@0.05 | 70.1% at 0.2 A $g^{-1}$ (500 cycles) | 93.1@1.0 | [10] |
| PPh-PTO | 1.5-3.8 V | 235@0.1 | 95 % at 0.1 A $g^{-1}$ (1400 cycles) | 94@2.0 | [11] |
| PTCOF50 | 1.5-3.5 V | 280@0.2 | 82 % at 2.0 A $g^{-1}$ (3000 cycles) | 229@5.0 | [12] |
| PPYT | 1.5-4.0 V | 231@1C | 83.5% at 1C (500 cycles) | 208@30 C | [13] |
| PPAPT | 2.0-4.0 V | 192@0.045 (1C) | 71.8% at 0.9 A $g^{-1}$ (500 cycles) | 156@0.9 (20C) | [14] |
| P(PTO-T1) | 1.5-3.5 V | 187@0.05 | 94 % at 2.0 A $g^{-1}$ (1200 cycles) | 72@2.0 | [15] |
| P(PTO-T2) | 1.5-3.5 V | 172@0.05 | 82 % at 2.0 A $g^{-1}$ (1200 cycles) | 105@2.0 | [15] |
| P(PTO-TT) | 1.5-3.5 V | 182@0.05 | 76 % at 2.0 A $g^{-1}$ (1200 cycles) | 111@5.0 | [15] |

| | | | | | |
|---|---|---|---|---|---|
| DE-PTO | 1.5-4.5 V | 296@0.15 | 165 % at 0.15 A $g^{-1}$ (1000 cycles) | 184@3.0 | [16] |
| PTO-Bz | 1.5-3.5 V | 195.2@0.1 | ~61.3 % mA h $g^{-1}$ at 1.0 A $g^{-1}$ (2000 cycles) | 138.3@10 | [17] |
| PTO-Py | 1.5-3.5 V | 220.4@0.1 | ~66.0 % at 1.0 A $g^{-1}$ (2000 cycles) | 153.7@10 | [17] |
| PTO-Pm | 1.5-3.5 V | 132.7@0.1 | ~74.9 % at 1.0 A $g^{-1}$ (2000 cycles) | 62.9@10 | [17] |
| PTO-Tz | 1.5-3.5 V | 205.3@0.1 | ~63.7% at 1.0 A $g^{-1}$ (2000 cycles) | 121.5@10 | [17] |
| **PTO-2CZ** | **1.5-4.5 V** | **202@0.2** | **72.7 % at 5 A $g^{-1}$ (2000 cycles)** | **119 @10** | **This work** |

**Table S2.** The reported donor-acceptor (D-A) electrodes in half battery.

| Organic cathode | Structure | Discharge voltage | Performance | Ref |
|---|---|---|---|---|
| TzPz | 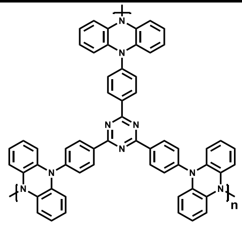 | 3.86, 3.11 V vs. Li/Li$^+$ | 192 mA h g$^{-1}$ at 0.2 A g$^{-1}$,<br>108 mA h g$^{-1}$ at 30 A g$^{-1}$,<br>10000 cycles at 5 A g$^{-1}$ | [18] |
| BPyPz | 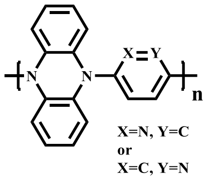 | 3.78, 2.97 V vs. Na/Na$^+$ | 205 mA h g$^{-1}$ at 0.5 C,<br>126 mA h g$^{-1}$ at 20 C,<br>1000 cycles at 10 C | [19] |
| TPyPz | 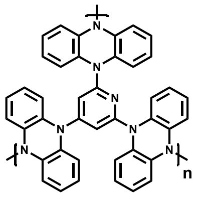 | 3.77 V vs. Na/Na$^+$ | 238 mA h g$^{-1}$ at 0.5 C,<br>66 mA h g$^{-1}$ at 20 C,<br>1000 cycles at 10 C | [19] |
| p-TTPZ | 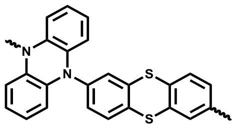 | 3.82, 3.16 V vs. Li/Li$^+$ | 152 mA h g$^{-1}$ at 0.1 A g$^{-1}$,<br>124.2 mA h g$^{-1}$ at 10 A g$^{-1}$,<br>1000 cycles at 2 A g$^{-1}$ | [20] |
| **PTO-2CZ** | 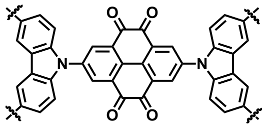 | **4.15, 2.87 V vs. Li/Li$^+$** | **202 mA h g$^{-1}$ at 0.2 A g$^{-1}$,<br>119 mA h g$^{-1}$ at 10 A g$^{-1}$,<br>2000 cycles at 5 A g$^{-1}$** | **This Work** |

**Table S3.** A comparison table includes several recently reported bipolar-type cathodes for lithium-ion batteries.

| **Organic cathode** | **Plateau voltage (V)** | **Reversible capacity (mA h $g^{-1}$@A $g^{-1}$)** | **Cycle life** | **Rate capacity @mAh $g^{-1}$@A $g^{-1}$)** | **Ref** |
|---|---|---|---|---|---|
| [Mn(phendi)$_3$]·2$PF_6$ | 3.0 | ≈150@0.0075 | N/A | N/A | [21] |
| Poly-DAAQ/FWNT | N/A | 311@0.05 | 100@1 | 241@1<br>197@5<br>134@10 | [22] |
| Cu-THQ MOF | 3.2-2.6 V/2.2-1.8 V/1.7-1.2 V | 387@0.05 | 100@0.05 | 159@0.5<br>93@1 | [23] |
| CTFs | N/A | 135@0.1 | 50@0.1 | 247@0.02<br>61@1 | [24] |
| poly-APCNDI | 4.0/2.4 | 141@0.1 | 1000@5 | 123@1<br>72@10 | [25] |
| PTB-DHZ-COF40 | > 3.6 V | 114.24@1 | 5000@1 | 114.24@1 | [26] |
| BNBQ | 3.77/2.56 | 133@0.1 | 5000@1 | 105@2 | [27] |
| Poly(CoL)$_n$ | 3.97/3.21/2.19 | 192.13@0.05 | 2000@5 | 104.1@5 | [28] |
| NT-PICOF | 3.93/3.54/2.36 | 165@0.03 | 4000@1 | 96@2 | [29] |
| CuPcNA-CMP | N/A | 202.4@0.15 | N/A | 86.1@5 | [30] |
| COP500-CuT2TP | 3.7/3.1/2.2/1.6 | 420@0.1 | 8000@5 | 86@10 | [31] |
| PVPTOCl$_2$ | 2.77/2.16/2.05 | 235@0.2 | 3000@1 | 150.3@5 | [32] |
| **PTO-2CZ** | **4.05/2.78** | **202@0.2** | **2000@5** | **119 @10** | **This work** |

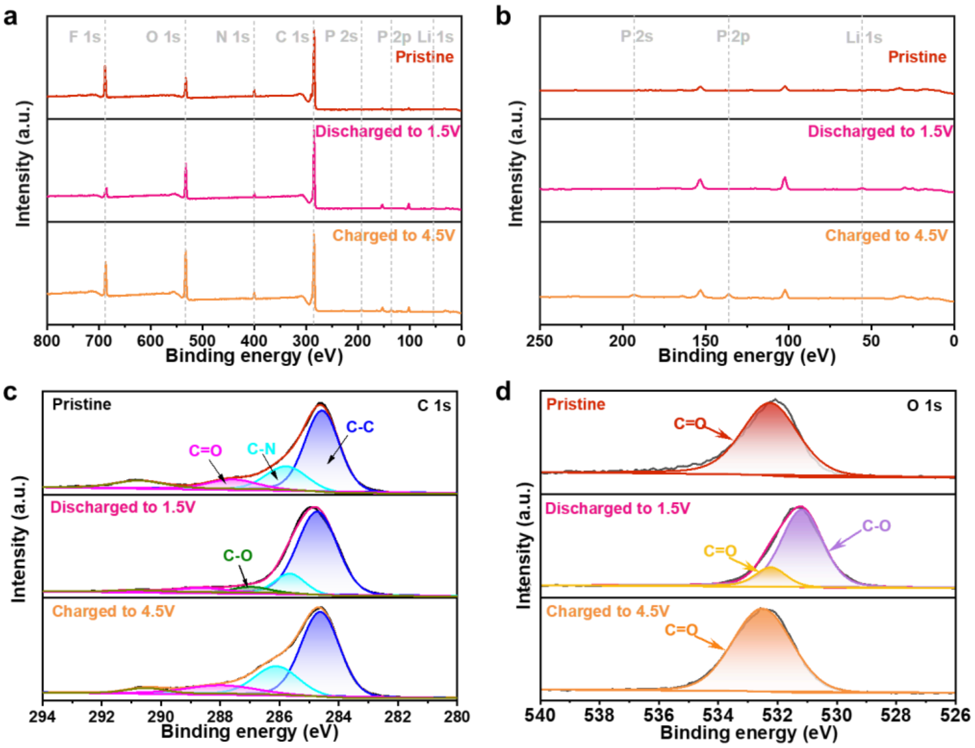


**Figure S14.** (a, b) Full survey spectra and (c) high-resolution C 1s spectra and (d) O 1s spectra of PTO-2CZ cathodes at different charge/discharge states.

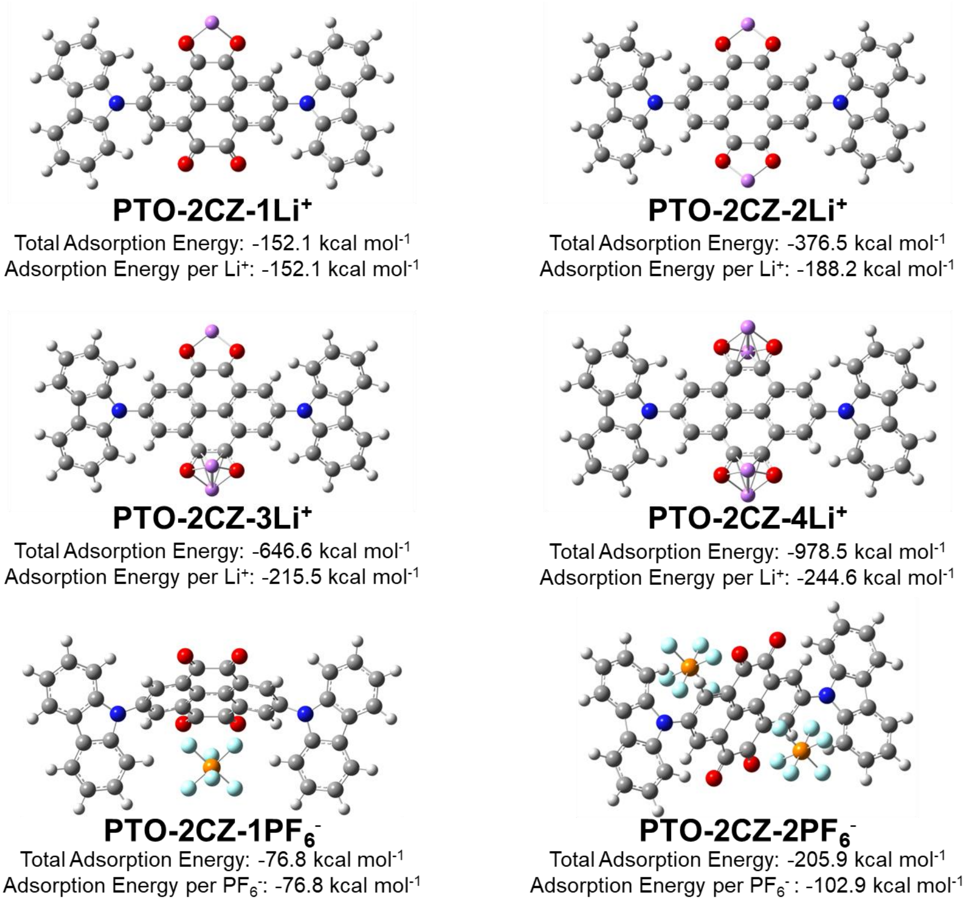


**Figure S15.** DFT-optimized structures, total adsorption energy and adsorption of PTO-2CZ at different cycled states.

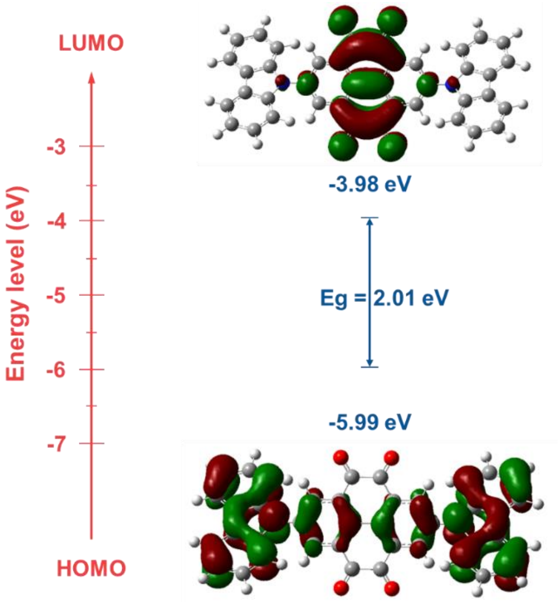


**Figure S16.** The HOMO/LUMO energy levels and orbital plots of PTO-2CZ; Eg is the HOMO-LUMO gap.

**Table S4.** NPA charges of PTO-2CZ-4Li and PTO-2CZ-$2PF_6$.

**NPA charges of PTO-2CZ-4Li**

| Atom | No. | NPA Charges |
|---|---|---|
| C | 1 | 0.1342 |
| C | 2 | -0.23397 |
| C | 3 | -0.07586 |
| C | 4 | -0.03517 |
| C | 5 | -0.07553 |
| C | 6 | -0.23385 |
| C | 7 | 0.15863 |
| C | 8 | -0.02862 |
| C | 9 | -0.07055 |
| C | 10 | 0.1403 |
| C | 11 | -0.2334 |
| H | 12 | 0.27115 |
| C | 13 | 0.13397 |
| C | 14 | -0.2301 |
| C | 15 | -0.08631 |
| C | 16 | 0.14487 |
| C | 17 | 0.15844 |
| H | 18 | 0.2711 |
| H | 19 | 0.2711 |
| H | 20 | 0.27116 |
| N | 21 | -0.39156 |
| C | 22 | 0.19611 |
| C | 23 | 0.17822 |
| C | 24 | -0.2774 |
| C | 25 | -0.09601 |
| C | 26 | -0.27301 |
| C | 27 | -0.06887 |
| C | 28 | -0.24002 |
| H | 29 | 0.25325 |
| C | 30 | -0.21178 |
| C | 31 | -0.24005 |
| H | 32 | 0.25317 |
| C | 33 | -0.21532 |

**NPA charges of PTO-2CZ-$2PF_6$**

| Atom | No. | NPA Charges |
|---|---|---|
| C | 1 | 0.23298 |
| C | 2 | -0.14215 |
| C | 3 | -0.06515 |
| C | 4 | 0.10439 |
| C | 5 | -0.07505 |
| C | 6 | -0.12759 |
| C | 7 | 0.50556 |
| C | 8 | 0.10397 |
| C | 9 | -0.07493 |
| C | 10 | 0.50479 |
| C | 11 | -0.12758 |
| H | 12 | 0.28256 |
| C | 13 | 0.23886 |
| C | 14 | -0.15625 |
| C | 15 | -0.05772 |
| C | 16 | 0.50537 |
| C | 17 | 0.50482 |
| H | 18 | 0.29226 |
| H | 19 | 0.28259 |
| H | 20 | 0.292 |
| O | 21 | -0.46123 |
| O | 22 | -0.46264 |
| O | 23 | -0.46266 |
| O | 24 | -0.46152 |
| N | 25 | -0.29177 |
| C | 26 | 0.15432 |
| C | 27 | 0.15592 |
| C | 28 | -0.2224 |
| C | 29 | -0.04078 |
| C | 30 | -0.1984 |
| C | 31 | -0.05241 |
| C | 32 | -0.23001 |
| H | 33 | 0.26978 |

| | | |
|---|---|---|
| C | 34 | -0.27388 |
| H | 35 | 0.24263 |
| H | 36 | 0.24314 |
| C | 37 | -0.27371 |
| H | 38 | 0.24264 |
| H | 39 | 0.24314 |
| H | 40 | 0.24324 |
| H | 41 | 0.24323 |
| N | 42 | -0.3916 |
| C | 43 | 0.19606 |
| C | 44 | 0.17818 |
| C | 45 | -0.27741 |
| C | 46 | -0.09601 |
| C | 47 | -0.27303 |
| C | 48 | -0.06888 |
| C | 49 | -0.24002 |
| H | 50 | 0.25325 |
| C | 51 | -0.21178 |
| C | 52 | -0.24006 |
| H | 53 | 0.25315 |
| C | 54 | -0.21532 |
| C | 55 | -0.27388 |
| H | 56 | 0.24263 |
| H | 57 | 0.24314 |
| C | 58 | -0.27372 |
| H | 59 | 0.24264 |
| H | 60 | 0.24314 |
| H | 61 | 0.24324 |
| H | 62 | 0.24323 |
| O | 63 | -0.99366 |
| O | 64 | -0.9935 |
| Li | 65 | 0.9432 |
| Li | 66 | 0.94306 |
| O | 67 | -0.99458 |
| O | 68 | -0.99474 |
| Li | 69 | 0.94297 |
| Li | 70 | 0.94282 |

| | | |
|---|---|---|
| C | 34 | -0.20369 |
| C | 35 | -0.22521 |
| H | 36 | 0.29566 |
| C | 37 | -0.20089 |
| C | 38 | -0.20703 |
| H | 39 | 0.26557 |
| H | 40 | 0.2583 |
| C | 41 | -0.20028 |
| H | 42 | 0.27296 |
| H | 43 | 0.2568 |
| H | 44 | 0.26013 |
| H | 45 | 0.25988 |
| N | 46 | -0.2917 |
| C | 47 | 0.1543 |
| C | 48 | 0.15592 |
| C | 49 | -0.22241 |
| C | 50 | -0.04079 |
| C | 51 | -0.19848 |
| C | 52 | -0.05243 |
| C | 53 | -0.23001 |
| H | 54 | 0.26978 |
| C | 55 | -0.2037 |
| C | 56 | -0.22523 |
| H | 57 | 0.29562 |
| C | 58 | -0.2009 |
| C | 59 | -0.20703 |
| H | 60 | 0.26557 |
| H | 61 | 0.2583 |
| C | 62 | -0.20028 |
| H | 63 | 0.27296 |
| H | 64 | 0.2568 |
| H | 65 | 0.26013 |
| H | 66 | 0.25988 |
| P | 67 | 2.75034 |
| P | 68 | 2.75034 |
| F | 69 | -0.62778 |
| F | 70 | -0.6004 |
| F | 71 | -0.60144 |

| | | |
|---|---|---|
| F | 72 | -0.63282 |
| F | 73 | -0.61038 |
| F | 74 | -0.64154 |
| F | 75 | -0.6418 |
| F | 76 | -0.6004 |
| F | 77 | -0.61038 |
| F | 78 | -0.62777 |
| F | 79 | -0.60144 |
| F | 80 | -0.6328 |

## 3. Reference


[1] Q. Li, H. Wang, H. g. Wang, Z. Si, C. Li, J. Bai, *ChemSusChem* **2020**, *13*, 2449-2456.

[2] S. Zheng, D. Shi, T. Sun, L. Zhang, W. Zhang, Y. Li, Z. Guo, Z. Tao, J. Chen, *Angew. Chem. Int. Ed.* **2023**, *62*, e202217710.

[3] Gaussian 16, Revision C.01, M. J. Frisch, G. W. Trucks, H. B. Schlegel, G. E. Scuseria, M. A. Robb, J. R. Cheeseman, G. Scalmani, V. Barone, G. A. Petersson, H. Nakatsuji, X. Li, M. Caricato, A. V. Marenich, J. Bloino, B. G. Janesko, R. Gomperts, B. Mennucci, H. P. Hratchian, J. V. Ortiz, A. F. Izmaylov, J. L. Sonnenberg, D. Williams-Young, F. Ding, F. Lipparini, F. Egidi, J. Goings, B. Peng, A. Petrone, T. Henderson, D. Ranasinghe, V. G. Zakrzewski, J. Gao, N. Rega, G. Zheng, W. Liang, M. Hada, M. Ehara, K. Toyota, R. Fukuda, J. Hasegawa, M. Ishida, T. Nakajima, Y. Honda, O. Kitao, H. Nakai, T. Vreven, K. Throssell, J. A. Montgomery, Jr., J. E. Peralta, F. Ogliaro, M. J. Bearpark, J. J. Heyd, E. N. Brothers, K. N. Kudin, V. N. Staroverov, T. A. Keith, R. Kobayashi, J. Normand, K. Raghavachari, A. P. Rendell, J. C. Burant, S. S. Iyengar, J. Tomasi, M. Cossi, J. M. Millam, M. Klene, C. Adamo, R. Cammi, J. W. Ochterski, R. L. Martin, K. Morokuma, O. Farkas, J. B. Foresman, and D. J. Fox, Gaussian, Inc., Wallingford CT, **2016**.

[4] T. Lu, Q. Chen, *Chemistry-Methods*, **2021**, *1*, 231-239.

[5] T. Lu, F. Chen, Multiwfn: *J. Comput. Chem.* **2012**, *33*, 580−592.

[6] W. Humphrey; A. Dalke; K. Schulten, VMD: Visual Molecular Dynamics J. Molec. Graphics **1996**, *14*, 33−38.

[7] J. Xie, W. Chen, G. Long, W. Gao, Z. J. Xu, M. Liu, Q. Zhang, *J. Mater. Chem. A* **2018**, *6*, 12985-12991.

[8] Q. Li, D. Li, H. Wang, H.-g. Wang, Y. Li, Z. Si, Q. Duan, *ACS Appl. Mater. Interfaces* **2019**, *11*, 28801-28808.

[9] C. J. Yao, Z. Wu, J. Xie, F. Yu, W. Guo, Z. J. Xu, D. S. Li, S. Zhang, Q. Zhang, *ChemSusChem* **2019**, *13*, 2457-2463.
[10] K. Li, Q. Li, Y. Wang, H.-g. Wang, Y. Li, Z. Si, *Mater. Chem. Front.* **2020**, *4*, 2697-2703.
[11] S. Zheng, L. Miao, T. Sun, L. Li, T. Ma, J. Bao, Z. Tao, J. Chen, *J. Mater. Chem. A* **2021**, *9*, 2700-2705.
[12] H. Gao, A. R. Neale, Q. Zhu, M. Bahri, X. Wang, H. Yang, Y. Xu, R. Clowes, N. D. Browning, M. A. Little, L. J. Hardwick, A. I. Cooper, *J. Am. Chem. Soc.* **2022**, *144*, 9434-9442.
[13] T. Nokami, T. Matsuo, Y. Inatomi, N. Hojo, T. Tsukagoshi, H. Yoshizawa, A. Shimizu, H. Kuramoto, K. Komae, H. Tsuyama, J. Yoshida, *J. Am. Chem. Soc.* **2012**, *134*, 19694-19700.
[14] S. Yeşilot, N. Kılıç, S. Sariyer, S. Küçükköylü, A. Kılıç, R. Demir-Cakan, *ACS Appl. Energy Mater.* **2021**, *4*, 12487-12498.
[15] X.-H. Chen, H. Lu, Z. Wu, H. Wang, S. Zhang, S. Mei, G. Long, Q. Zhang, C.-J. Yao, *J. Mater. Chem. A* **2023**, *11*, 77-83.
[16] H. Pan, Z. Zuo, F. He, Y. Li, *Energy Storage Mater.* **2022**, *52*, 465-472.
[17] Y. Li, Y. Ding, J. Wu, Y. Wang, L. Chen, Y. Jin, L. Zhang, S.-B. Ren, D.-M. Han, *J. Mater. Chem. A* **2023**, *11*, 22813-22821.
[18] W. Ma, L. W. Luo, P. Dong, P. Zheng, X. Huang, C. Zhang, J. X. Jiang, Y. Cao, *Adv. Funct. Mater.* **2021**, *31,* 2105027.
[19] J. Zhang, H. Liu, K. Jia, X. Li, X. Liu, L. Zhu, R. He, F. Wu, *J. Mater. Chem. A* **2023**, *11*, 2711-2717.
[20] M. Fu, Y. Chen, W. Jin, H. Dai, G. Zhang, K. Fan, Y. Gao, L. Guan, J. Chen, C. Zhang, J. Ma, C. Wang, *Angew. Chem. Int. Ed.* **2023**, *63,* e202317393.
[21] A. E. Lakraychi, S. De Kreijger, D. Gupta, B. Elias, A. Vlad, *ChemSusChem* **2020**, *13*, 2225-2231.
[22] T. Liu, K. C. Kim, B. Lee, S. Jin, M. J. Lee, M. Li, S. Noda, S. S. Jang, S. W. Lee, *ACS Appl. Energy Mater.* **2020**, *3,* 3728-3735.
[23] Q. Jiang, P. Xiong, J. Liu, Z. Xie, Q. Wang, X. Q. Yang, E. Hu, Y. Cao, J. Sun, Y. Xu, L. Chen, *Angew. Chem. Int. Ed.* **2020**, *59*, 5273-5277.
[24] Z. Wang, S. Gu, L. Cao, L. Kong, Z. Wang, N. Qin, M. Li, W. Luo, J. Chen, S. Wu, G. Liu, H. Yuan, Y. Bai, K. Zhang, Z. Lu, *ACS Appl. Mater. Interfaces* **2020**, *13*, 514-521.
[25] W. Wang, C. Zhao, J. X. Yang, P. X. Xiong, H. Su, Y. H. Xu, *Sci. China Mater.* **2021**, *64*, 2938-2948.
[26] W. Li, Q. Huang, H. Shi, W. Gong, L. Zeng, H. Wang, Y. Kuai, Z. Chen, H. Fu, Y. Dong, C. Zhang, *Adv. Funct. Mater.* **2023**, 2310668.
[27] L. Zheng, J. Ren, H. Ma, M. Yang, X. Yan, R. Li, Q. Zhao, J. Zhang, H. Fu, X. Pu, M. Hu, J. Yang, *J. Mater. Chem. A* **2023**, *11*, 108-117.
[28] C.-x. Zhang, X.-h. Chen, W.-s. Zhang, Y. Wang, S.-l. Mei, Y.-W. Zhong, C.-J. Yao, *Chem. Eng. J.* **2024**, *483,* 149198.
[29] S. Gu, J. Chen, R. Hao, X. Chen, Z. Wang, I. Hussain, G. Liu, K. Liu, Q. Gan, Z. Li, H. Guo, Y. Li, H. Huang, K. Liao, K. Zhang, Z. Lu, *Chem. Eng. J.* **2023**, *454,* 139877.

[30] H. g. Wang, Q. Li, Q. Wu, Z. Si, X. Lv, X. Liang, H. Wang, L. Sun, W. Shi, S. Song, *Adv. Energy Mater.* **2021**, *11*, 2100381.
[31] X. Wu, W. Zhou, C. Ye, J. Zhang, Z. Liu, C. Yang, J. Peng, J. Liu, P. Gao, *Angew. Chem. Int. Ed.* **2024**, e202317135.
[32] A. Yu, C. Li, X. Chen, C. Zhang, S. Mei, C. J. Yao, *ChemSusChem* **2024**, *17*, e202301809.
